# Supplementary material for: Are carbon emissions trading and green financial instruments synergistic? -Comprehensive quantitative research based on content analysis
Source: PLoS One. 2024 Mar 7;19(3):e0298601. doi: 10.1371/journal.pone.0298601 (PMC10919601; doi:10.1371/journal.pone.0298601)
Supplement: S1 Data — (ZIP) [file pone.0298601.s001.zip › Data/地级市Z_F工作报告.zip!/╣π╬≈/╟╒╓▌╩╨/└┤╫╘╣1⁄2╓┌║┼í╛┬φ┐╦╩2╛▌═°í┐_2011.docx]

各位代表：

现在，我代表市人民政府向大会报告工作，请各位代表连同《钦州市国民经济和社会发展第十二个五年规划纲要（草案）》一并审议，并请市政协委员和其他列席会议的同志提出意见。

一、“十一五”时期经济社会发展回顾

“十一五”是我市建市以来发展最快最好的时期，也是蓄势发展的五年。五年来，党中央、国务院和自治区党委、政府高度重视钦州的发展，温家宝、贾庆林、李长春、回良玉、刘云山、刘延东、张德江等党和国家领导人亲临钦州视察指导，自治区提出钦州要成为广西科学发展的排头兵、跨越发展的先行区、改革开放的试验区，为钦州经济社会发展开辟了广阔前景。在中国－东盟自由贸易区建成、新一轮西部大开发实施及广西北部湾经济区开放开发的新形势下，中央还赋予钦州保税港区、整车进口口岸等一系列特殊政策，为钦州打造沿海经济新增长极提供了强大动力，钦州面临千载难逢的跨越发展机遇。在市委的坚强领导下，在市人大、市政协的全力支持下，全市人民同心同德抢抓机遇，齐心协力战胜危机，开拓创新破解难题，胜利完成“十一五”规划目标任务，全市经济社会发展跃上了新的历史起点！

——这五年，是发展地位大跃升的五年。广西北部湾经济区开放开发上升为国家战略，中石油2000万吨炼油厂、2000万吨储备油库、林浆纸一体化等支撑性项目落户钦州，获批钦州保税港区、整车进口口岸、国家级经济技术开发区等“国”字号金字招牌，极大提升了钦州的品牌效应，使钦州从对外开放的“末梢”走向最前沿，在打造区域合作新高地和构筑沿海发展新一极的历史使命中发挥重要的作用，成为支撑和带动西部大开发的战略平台。钦州在对外开放与合作中的优势地位更加凸显、加快钦州跨越发展的信心和愿望更足更强！

——这五年，是综合实力大提升的五年。2010年与2005年相比，地区生产总值、人均生产总值、工业总产值、社会消费品零售总额、金融机构存款余额、金融机构贷款余额等六个指标翻一番以上，财政收入、固定资产投资、港口吞吐能力、港口吞吐量、外贸进出口、利用外资、引进国内到位资金等七个指标翻两番以上。据初步统计，2010年地区生产总值完成504.18亿元，年均增长16.1%，增速比“十五”高5.3个百分点；财政收入完成58.37亿元，年均增长32.8%，增速比“十五”高19.3个百分点；工业总产值593.59亿元，年均增长27%，增速比“十五”高5.2个百分点；全社会固定资产投资451.6亿元，年均增长38.1%，五年累计超过1300亿元，相当于建市以来至“十五”末期的3.5倍。产业结构调整实现重大突破，三次产业比例由40.7∶27.4∶31.9调整为26.2∶40.8∶33，工业增加值占地区生产总值的比重从22.2%跃升至34.7%，超过了农业的比重，实现了经济发展方式由农业为主向以工业为主导的转变，综合实力由全区下游水平提升到中游水平。

——这五年，是港工城联动大发展的五年。坚持以港口为龙头，产业为支撑，城市为依托，形成了港口、工业、城市良性互动发展的格局。一是亿吨大港加快建设。填海造地2710公顷，演绎了“精卫填海”的神奇故事，保障了重大项目用海用地，改写了钦州版图。10万吨级航道等一批港口基础设施项目建成使用，开工建设30万吨级航道和油码头等一批深水码头、航道，以及总投资133.4亿元、总长190公里的3条高速公路和总投资117.5亿元、总长189公里的7条铁路。加密通往香港等地的集装箱班轮航线，增开钦州至高雄、钦州至台北、钦州至海防以及南北直航等集装箱航线，出海出边通道优势逐步形成。港口吞吐能力新增4297万吨，达到5346万吨；集装箱吞吐量突破25万标箱。二是临港产业迅速崛起。中石油广西石化一期1000万吨炼油厂和原油储备库一期420万方库区、国投燃煤电厂一期、金桂林浆纸制浆等一批重大项目建成投产，中粮120万吨大豆加工、清华同方数字电视、玉柴石化、泰兴石化、锐丰钒钛铁等一批重大产业项目开工，初步形成了石化、造纸、电子、能源、粮油食品、冶金、物流七大产业。2010年，钦州港经济开发区规模以上工业总产值达到288.24亿元，占全市比重的59.6%。三是城镇化步伐加快。城市总规修编完成，河东新区加快拓展，“城乡风貌改造”、“城乡清洁工程”和“园林生活十年计划”全面实施，以“一环六横六纵七通道”为主干道的城市路网加快建设，城市供水、供电、污水垃圾处理等城市基础设施进一步完善。城镇化率提高9个百分点达到35.5%，中心城区面积扩大20平方公里，达到68.7平方公里，绿化覆盖率达到31.5%，供水能力达到24.2万吨/日，污水垃圾处理设施实现市县全覆盖。三娘湾、刘冯故居、八寨沟先后评定为国家4a级景区，灵山县成为“广西优秀旅游县”，钦州成为“中国优秀旅游城市”、第七届中国－东盟博览会中国“魅力之城”。

——这五年，是改革开放成果大丰收的五年。坚持规划先行，基本完成城市、产业、交通和民生四大类42项规划编制工作。国企改制和投融资平台建设取得新突破，“国企改制三年攻坚”完成203家企业改制，盘活国有资产43.7亿元；整合组建资产达190.9亿元的市开投集团。“引银入钦”和金融创新成效显著，新引进五家银行，率先在全区开展信托融资7.93亿元、金融租赁2亿元，成功发行企业债券8亿元；积极开展离岸金融业务试点和跨境贸易人民币结算试点。创新征地拆迁和用地用海机制，建立“征、拆、储、建、安、保”一体化的征地拆迁机制，重点推行公寓式安置，开展三个区域26平方公里的用海整体报批试点。积极创新人才使用机制，组建港口物流发展、产业发展、新城建设和发展、区域合作与发展四个战略领导小组和专家咨询组，出台引进高层次人才的系列政策措施，在全区率先推行政府雇员试点。实施科技创新计划取得新成果，建成国家级石油化工品检测重点实验室（广西钦州）、国家石化产品质量监督检验中心（钦州）等质量监督检验中心。林权制度改革走在全区前列，政府机构改革、医药卫生体制改革等各项改革迈出新步伐。

——这五年，是改善民生构建和谐取得大进展的五年。坚持每年为民办实事，全市各级财政在教育、卫生、社会保障和就业、文化等八类民生领域投入累计达到162.78亿元，是“十五”的4.46倍，年均增长40.8%。实施“就业、创业、兴业”工程，五年新增农村劳动力转移就业26万人，新增城镇就业8.4万人，城镇登记失业率每年都在4%以内。解决82万农村人口的饮水安全问题，建成一批保障性住房。实现100%行政村通等级公路，69%行政村通水泥路。“村村通”广播电视工程完成3908个村点，覆盖农村人口44万人。社会保障“五险”市级统筹走在全区前列，养老、医疗、失业、工伤和生育保险参保人数达70.55万人次，比“十五”期末增加44.23万人次；新型农村合作医疗农民参合率达到92.4%；23.99万农村居民参加新型农村养老保险。3.9万城市困难居民和19.5万农村困难农户享受最低生活保障，基本实现动态下的应保尽保。扶贫开发整村推进、新农村建设成效显著。城镇居民人均可支配收入年均增长14.2%，农民人均纯收入年均增长11.6%，居民消费价格年均涨幅基本稳定在3.5%。

坚持教育优先发展，巩固提升“两基”成果，普及九年义务教育，大力发展高中教育，建成市一中及市三中新校区、子材小学，扩建市八小等学校；积极发展职业教育和高等教育，建成市职业教育中心一期、广西英华国际职业技术学院，钦州学院实现专升本，积极申办北部湾大学。成功申办广西第十二届运动会和广西第十三届少数民族传统体育运动会，基层群众文化生活进一步改善，文化遗产保护工作得到加强。民间舞蹈《纵情岭头》、海歌剧《豪叔与秀姑》等一批文艺作品在全国全区获奖。民主法制建设得到加强，社会治安综合治理不断强化，荣获全区“平安先进市”。环境保护和生态建设扎实推进，全面完成“十一五”节能减排目标，荣获“中国节能减排20佳城市”；耕地保护连续5年考核合格，生态保持良好。建立健全计划生育利益导向机制，人口自然增长率控制在10‰以内。

——这五年，是不断完善发展思路、探索积累成功经验的五年。五年来，钦州发展的思路越来越清晰，全面实施了市委提出的“大港口、大工业、大旅游”、“以港兴工，三化互动”和“建大港、兴产业、造新城”发展方略，开展“三大会战攻坚年”、“重大项目建设年”、“千百亿产业崛起工程建设年”、“服务企业年”、“工作落实年”等活动。克服了百年一遇的全球金融危机、冰冻雨雪灾害等不利影响，坚持加快发展不动摇，凝心聚力，攻坚破难，发展的步伐越来越坚定，发展的前景越来越广阔！

五年积累的经验弥足珍贵。我们深深体会到：必须坚持以科学发展观为统领，勇于探索推动跨越发展的新思路；必须坚持越是后发展越要坚持规划先行，越是后发展越要重视生态环保；必须抢抓机遇，把抓投资上项目作为加快发展的重要抓手，以超常规措施加快经济社会发展，不断增强经济发展后劲；必须把改革开放作为跨越发展的强大动力，着力破解发展瓶颈，积极融入多区域合作；必须坚持以人为本，把保障和改善民生作为工作的出发点和基本要求，促进经济社会协调发展；必须强化行政效能和政府自身建设。五年来形成的发展基础、积累的宝贵经验、创造的精神财富必将对钦州未来发展产生深远影响，为钦州发展插上腾飞的翅膀！

刚刚过去的2010年，是钦州发展进程中具有重大意义的一年。面对严峻复杂的经济形势，全市上下深入贯彻落实科学发展观和中央、自治区的一系列决策部署，围绕重大项目加快建成、重要平台加快见效这一目标，以“建大港、兴产业、造新城”为引擎，以“攻坚、服务、收获”为主线，开拓进取，干事创业，全面完成了三届人大六次会议确定的目标任务，为“十一五”划上了圆满句号。

过去的一年，我们干成了四件具有里程碑意义的好事大事：一是钦州港经济开发区升格为国家级经济技术开发区，钦州成为全区仅有的两个拥有国家级经济技术开发区的城市之一；二是钦州港口岸扩大对外开放范围获国家批准，新增开放岸线28.776公里、泊位98个；三是北部湾经济区开放开发标志性工程中石油千万吨炼油厂、金桂林浆纸制浆工程建成投产；四是滨海新城建设全面启动。

过去的一年，我市经济社会发展实现了七大突破。一是全市生产总值突破500亿元，比上年增长18%，增速排全区第一，创建市以来最高水平；二是财政收入突破50亿元，增长53.5%，增速排全区第一；三是工业总产值突破500亿元，增长63.3%，增速排全区第一；四是港口吞吐量突破3000万吨，达到3022万吨，其中集装箱吞吐量突破25万标箱，是2009年的2.5倍，跃居全区沿海港口首位；五是招商引资实现新突破，引进区外国内到位资金320亿元，总量居全区第三位，实际利用外资3.2亿美元，总量居全区第二位；六是“引银入钦”实现新突破，金融机构本外币存款余额476.15亿元，贷款余额326.55亿元，分别比年初增长28%和29.7%，增速位居全区前列；七是征地拆迁实现了公寓式安置和失地农民养老保险工作的新突破。同时，各项社会事业协调发展，人民群众得到了更多实惠。

一年来，我们主要做好以下几个方面的工作：

（一）抓项目促投资，推动经济持续快速增长。扎实开展“重大项目攻坚年”活动，建立完善市领导联系重大项目制度、投资和项目建设定期会商制度，推行联合集中审批常态化，组织项目集中开竣工，抽调精干力量开展征地拆迁攻坚活动，促进项目落地和建设。全市投资500万元以上项目772个，同比增加33个，其中亿元以上项目122个，同比增加24个。全年集中开竣工项目167个，总投资578亿元。争取自治区追加建设用地指标21428亩，相当于年初下达指标的3.8倍。

（二）抓攻坚重服务，促进工业经济加快崛起。成功完成了中石油1.2公里环境敏感区居民和谐大搬迁，积极协调解决林浆纸一体化项目银团贷款闭合问题，确保两大项目竣工投产。国内最大的锰矿烧结项目东方资源60万吨锰烧结、灵山桂合丝业等相继建成投产。中粮120万吨大豆加工等项目加快建设，引进中海洋300万吨沥青、阿帕奇电动汽车、香港锐丰钒钛铁等一批产业项目。扎实推进“县域工业突破工程”，年内新增规模以上工业企业87家，其中县域工业企业占了86家。规模以上工业总产值483.31亿元，比上年增长78.9%，增幅居全区第一位。

（三）建通道畅物流，加快建设南方亿吨大港。推进深水航道和大能力泊位建设，加快构建完善港口集疏运体系，南宁至钦州等七条铁路和钦州港至六景等两条高速公路加快建设。大力培育集装箱物流，加密和开通通往香港、台北、海防等地的集装箱班轮航线，全面推行口岸联检部门“一站式”服务，通关效率和服务水平进一步提高。开展5亿吨大港的规划研究。基本完成区域性国际航运中心发展规划。

（四）改风貌造新城，加快建设宜商宜居城市。高标准完成110平方公里的滨海新城规划和茅尾海综合整治规划，启动45平方公里的滨海新城开发建设，规划建设保税港区新城、三娘湾旅游度假区，开工建设钦州港行政商务中心。加快“一环六横六纵七通道”城市路网建设，体育场馆主体工程基本完成，稳步推进城市管网、污垃处理等城市基础设施建设。开展宾馆升星改造，完成市区客运三轮车整治优化工作；深化拓展“城乡清洁工程”，积极参与市容“南珠杯”竞赛；实施“园林生活十年计划”和“城乡风貌改造”。加快灵山县城、浦北县城和钦南区、钦北区新城区建设，推动重点街区、重点城镇规划建设。

（五）稳三农活商贸，统筹城乡协调发展。认真落实种粮、良种、农机具购买、家电下乡补贴等一系列强农惠农政策，稳定粮食生产，实现总产109万吨。推行农业标准化生产，新增一批无公害农产品产地。抓好大宗农产品的流通服务工作，荔枝、龙眼、香蕉、马铃薯等大宗农产品实现顺产畅销。强化重大动物疫病防控，有效防止大的疫病疫情发生。加快推进病险水库、大型灌区渠道、农村人饮、标准海河堤等农田水利工程建设，解决32万农村人口饮水安全问题。新增植树造林面积7900公顷，新增和恢复灌溉面积4.5万亩。加强农村金融服务，组建灵山县泰业村镇银行和浦北县民兴小额贷款公司。新增农民专业合作经济组织72家，新增年产值5000万元以上龙头企业9家。

加快广西北部湾（钦州）台湾农民创业园、中国－东盟（钦州）农产品大市场等专业市场规划建设，深化与东盟国家农业合作；完善农村物流服务体系，推进“万村千乡市场工程”、“双百市场工程”、“放心肉工程”等建设。积极开拓城乡消费市场，培育新的消费热点，扩大消费规模；年内社会消费品零售总额172.19亿元，增长18.8%。

（六）保民生促和谐，社会事业全面进步。在抓好经济发展的同时，我们更加注重社会管理和民生改善。关系民生的八类支出51.8亿元，占一般预算支出的64.5%。医药卫生体制改革顺利启动， 60%以上的基层医疗卫生机构实施国家基本药物制度，缓解了群众“看病难、看病贵”问题。为民办10件实事全面完成，就业和劳动保障得到加强，安全生产形势稳定，社会治安综合治理进一步加强，扎实开展“落实科学发展观千名干部下访回头看”活动，积极推进社会矛盾化解、社会管理创新、公正廉洁执法三项重点工作，不断完善应急管理体系，有效维护社会稳定。加强生态环保建设，全面完成节能减排目标任务。

过去的一年，国防动员和后备力量建设进一步加强，双拥优抚工作进一步落实，军民团结关系得到巩固。海关、出入境检验检疫、边检、海事、口岸以及驻北京联络处等在钦州对外开放中发挥了重要作用。税务、人民银行、银监、工商、质监、电力、食品药品监督、烟草、通信、邮政、盐务等在服务地方发展中作出了贡献；民族、宗教、外事、侨务、统计、监察、审计、物价、农机、供销、气象、地震、水文、人防、海防、新闻出版、广播电视、档案、修志等工作稳步推进，妇女儿童、老龄、残疾人等事业取得新进展。

各位代表，回顾过去的五年，我们欣喜地看到，钦州的经济更加繁荣，城市更加美丽，人民生活更加殷实，社会更加和谐。这些成绩的取得，是自治区党委、政府正确领导的结果，是历届党委、人大、政府、政协打下坚实基础和各民主党派、工商联支持帮助的结果，是全市各族人民团结奋斗、顽强拼搏的结果。在此，我代表市人民政府，向为钦州发展付出辛勤劳动的全市各族人民致以亲切的问候和崇高的敬意！向所有关心、支持钦州发展的同志们、朋友们，向驻钦中直区直单位、驻钦部队、武警官兵表示衷心的感谢！

在肯定成绩的同时，我们也清醒地认识到，经济社会发展中还面临不少困难和问题，后发展欠发达的市情仍未根本改变。主要体现在：缺乏后续重大项目的支撑，投资对经济增长的拉动力后劲不足；资金短缺、征地拆迁等仍是困扰我市经济发展的瓶颈问题；农村基础设施建设滞后；节能减排任务艰巨，生态环保任重道远；关系群众切身利益的就业、住房、医疗、教育等问题有待更好解决，维护社会稳定压力继续增大；政府社会管理和公共服务水平还不高，执行力有待增强。

为此，我们一定要增强忧患意识和责任感，以更加昂扬的斗志，更加奋发有为的精神状态，更加扎实的工作作风，努力把政府各项工作做得更好，绝不辜负人民群众对更加美好生活的期盼！

二、“十二五”时期经济社会发展的主要目标和任务

今后五年，是我市同步建成小康社会的重要阶段，是我市加快转变经济发展方式、从蓄势发展进入跃升发展的关键时期。根据中共钦州市委《关于制定钦州市国民经济和社会发展第十二个五年规划的建议》（以下简称《建议》），市政府认真研究并听取各方面意见，制定了《钦州市国民经济和社会发展第十二个五年规划纲要（草案）》，提请大会审议。下面我简要作个说明。

根据《建议》，“十二五”时期的指导思想是：高举中国特色社会主义伟大旗帜，以邓小平理论和“三个代表”重要思想为指导，深入贯彻落实科学发展观，大力实施西部大开发战略，牢牢把握北部湾经济区开放开发上升为国家战略的历史机遇，紧紧围绕实现“富民强桂”新跨越，以科学发展为主题，以加快转变经济发展方式为主线，以“建大港、兴产业、造新城、强科教、惠民生”为发展方略，以“十大工程”为抓手，进一步深化改革，全方位融入国际区域开放合作，着力使经济更具活力，着力使城市更有魅力，着力使社会更加和谐，着力使群众生活得到更大改善，努力把钦州建设成为区域性国际航运物流枢纽、产业合作枢纽、市场交易枢纽和宜商宜居海湾新城，为全面建成小康社会打下具有决定性意义的基础。

根据上述指导思想，未来五年我市经济社会发展的主要目标和任务是：

——经过五年努力，经济总量跃居广西前列，结构调整上新台阶。2015年，实现地区生产总值达1200亿元（按当年价格计算）以上，五年平均增长 13%左右；人均生产总值超过全区平均水平；财政收入达到165亿元，年均增长23%左右；全社会固定资产投资累计完成3000亿元，年均增长15%。地区生产总值、财政收入、全社会固定资产投资、工业总产值、社会消费品零售总额、港口吞吐能力和吞吐量、外贸进出口总额、利用外商直接投资、引进国内资金翻一番以上，主要经济指标排位前移、跃居全区前列，进入广西中上水平。三次产业结构调整为17∶55∶28，工业增加值占地区生产总值的比重超过50%，钦州步入以工业为主导的工业化中期阶段。

——经过五年努力，实现亿吨大港目标，基本形成集装箱干线港。以实施亿吨大港工程、东盟商贸物流基地工程为主要抓手，大力推进深水航道和大能力泊位建设，完善出海通边、服务“三南”的集疏运体系和航运服务体系，全面建成钦州保税港区、整车进口口岸，为把钦州港建设成为面向中国－东盟合作的区域性国际航运中心和物流中心打下坚实基础。到2015年，钦州港迈进全国亿吨大港行列，确立集装箱干线港地位，港口吞吐能力达到1.2亿吨以上，其中集装箱500万标箱；港口吞吐量完成8000万吨，其中集装箱200万标箱；力争港口吞吐量实现1亿吨，其中集装箱250万标箱。

——经过五年努力，北部湾临海核心工业区初步建成，形成富有竞争力的现代产业体系。围绕工业强市目标，深入实施千百亿产业崛起工程、县域工业突破工程、高新产业培育工程，加快构建园区经济体系建设，快速壮大临港产业集群，积极发展装备制造业，大力培育战略性新兴产业，优化提升特色农业，加快发展现代服务业，积极发展海洋经济，推动产业结构优化升级，不断提升产业核心竞争力。2015年，工业总产值达到2300亿元，年均增长31%，研究与试验发展经费支出占地区生产总值比重达到1.1%。打造1至2个产值超千亿元、7个以上超百亿元产业，培育5个以上产值超百亿元企业，建成一个产值超千亿元的产业园区，成为引领北部湾经济区沿海产业发展的龙头。

——经过五年努力，宜商宜居海湾新城初具规模，城市的辐射带动作用更加突出。按照“东进南拓、向海发展”的空间布局，以滨海新城建设为龙头，积极实施滨海新城开发工程、“园林生活十年计划”生态建设工程，不断优化提升城市功能和城市管理水平。加强与东盟城市合作，主动对接首府南宁，加快发挥南（南宁）钦（钦州）同城效应，密切与东西两翼兄弟城市联系合作，积极服务北部湾城市群发展。加快县城和重点镇建设，提高城镇化水平。到2015年，建成滨海新城起步区，基本形成滨海新城、主城区、钦州港区、三娘湾旅游度假区“一城三区”城市组团格局，创建成为国家级园林城市。全市城镇化率力争达到50%左右。

——经过五年努力，区域经济合作提升新水平，形成更具活力的发展机制。深化以东盟为重点的国内外区域合作，提升钦州对外开放的新优势；积极兴办产业合作区，推进加工贸易集聚，拓展对外产业合作空间；全方位招商引资，大力引进央企、外企、民企，加快形成“大招商、招大商”的氛围；扩大对外贸易，调整优化外贸产品结构，扩大外贸规模，推进出口市场多元化。钦州在面向东盟的航运物流、产业对接、市场交易、文化交流中发挥更大的作用。2015年，外贸进出口总额突破100亿美元，年均增长50%；五年累计利用外资26亿美元，累计引进国内到位资金2500亿元。

——经过五年努力，人民生活更加殷实。加快推进农业现代化，推动城乡一体化发展。加强社会管理创新，积极实施城镇保障性住房安居工程，健全覆盖城乡居民特别是失地农民、失海渔民的社会保障体系，提高社会保障水平。推进基本公共服务均等化，提高医疗卫生服务能力，提高人口服务水平。实施“三业行动”富民工程，拓宽就业增收渠道，合理调整收入分配，帮助人民群众过上更好生活。“十二五”期间，城镇居民人均可支配收入、农民人均纯收入年均增长10%，实现城乡居民收入赶超全国平均水平；城镇登记失业率控制在4.5%以内，五年城镇新增就业人数10万人。2015年，城乡三项医疗保险参保率提高到95%以上；城镇基本养老保险参保人数13.2万人。

——经过五年努力，社会更加安全稳定和谐。大力实施科教和人才强市工程，推动科技创新，强化人才保障。实施教育发展10大重点工程和推进教育体制10大改革试点，学前三年毛入园率达到61%，义务教育巩固率96%。加强社会主义民主法制和精神文明建设，推进平安和谐钦州建设。以承办广西第十二届运动会和广西第十三届少数民族传统体育运动会为契机，加快应急指挥体系建设，提高突发事件处置能力。以创建全国文明城、国家卫生城和国家园林城为载体，提高全民文明素质。建设青少年活动中心、妇女儿童活动中心、图书馆、北部湾博物馆等一批项目，积极发展公益性文化事业，繁荣经营性文化产业，推进文化创新，促进文化大发展大繁荣。

——经过五年努力，生态文明建设全面提升。加快资源节约型和环境友好型社会建设，大力发展循环经济、低碳经济，倡导绿色消费理念；强化能源资源节约利用，加强生态环境保护和防灾减灾体系建设，全面完成国家和自治区下达的节能减排目标任务，做到既要创造金山银山，更要保住绿水青山。2015年，耕地保有量保持在345万亩左右，森林覆盖率达到55%，森林蓄积量增加到0.22亿立方米。

各位代表，全面实现“十二五”发展的宏伟目标，任务艰巨而光荣。我们坚信，勤劳智慧的钦州人民，必将继续发扬“钦州精神”，奋发有为，勇于超越，争创一流，谱写钦州跨越发展的新篇章！

三、努力实现“十二五”良好开局

今年是中国共产党成立90周年，是实施“十二五”规划的第一年。我们必须只争朝夕，克难攻坚，全力以赴做好今年的各项工作，努力为“十二五”开好局、起好步。

今年我市经济和社会发展的主要预期目标建议是：生产总值增长16%；财政收入增长54%；全社会固定资产投资增长20%，其中技改投资增长25%；规模以上工业总产值增长52%；社会消费品零售总额增长16%；城镇化率达到36.5%；港口吞吐量4000万吨，其中集装箱吞吐量40万标箱；外贸进出口总额增长52%，其中出口总额增长22%；实际利用外资增长25%，引进国内到位资金增长25%；城镇居民人均可支配收入、农民人均纯收入分别增长10%；居民消费价格上涨控制在5%左右；万元生产总值能耗、化学需氧量和二氧化硫排放总量等控制在自治区下达的目标之内；城镇新增就业1.75万人，城镇登记失业率和人口自然增长率分别控制在3.5%和10‰以内。

在实际工作中，我们将朝着更高的目标努力，保持干事创业的韧劲、冲劲和闯劲，再创钦州发展新局面！

（一）坚定不移建设亿吨大港，提升港口功能和竞争力

全力推进深水航道和大能力泊位建设。加强与北部湾国际港务集团、北部湾投资开发集团、广西交通投资集团、广西铁路投资集团的战略合作，加快建设30万吨级航道及油码头、金鼓江航道。开工建设保税港区9#－11#泊位、大榄坪北1#－3#泊位，基本建成保税港区3#－8#泊位、12#－13#油码头泊位，全面启动大榄坪综合物流加工区10.08平方公里的吹填工程。开展钦州港物流模型实验和港口辅助体系布局研究，基本完成5亿吨大港规划研究和钦州航运服务集聚区规划、锚地规划等。加快20万吨级航道及矿石码头等一批项目的前期工作。加强海上防灾减灾应急保障体系建设。

加强集疏运体系建设。加快构建以高速铁路、高速公路为骨架，以普通铁路干、支线，高等级公路为网络，以农村公路为补充，以站场枢纽为节点，包括管道、航空等多种方式为一体的交通运输体系。重点推进“七铁四高”和滨海公路、钦灵一级公路等一批铁路、公路建设。抓好市区交通主枢纽客运中心等一批站场建设，加快推进火车东站—大榄坪—三墩铁路、火车东站综合客运枢纽站场、龙门跨海大桥等一批项目的前期，争取尽快开工。启动城市轻轨、航空通用机场和基地的规划研究。加快推进灵山沙坪港码头建设和航道疏浚工程。

大力培育港口物流市场。以港口综合物流加工区为载体，推进与国内外知名港航物流企业合作，开拓加密航班航线，积极推进无水港建设，加快发展腹地外贸物流。争取建设钦州港铁路集装箱办理站和开通海铁联运定点班列。加大扶持力度，鼓励外贸集装箱向钦州保税港集聚。

全力加快航运服务体系建设。建设航运服务区，推动口岸基础设施和信息化建设，开工建设钦州港口岸联检大楼，进一步完善大通关机制。加快发展航运金融服务。积极协助自治区加快推进平陆运河前期工作。

全力服务好钦州保税港区建设运营。全力支持服务好保税港区全面开港运营，加快推进二、三期工程和整车进口口岸建设，推进汽车滚装码头、集装箱泊位、整车进口展示交易中心、国际酒类交易中心等项目加快建设，积极探索发展保税期货、离岸金融等业务。

（二）坚定不移抓项目促投资，做大工业规模，优化产业结构

深入实施“千百亿产业崛起工程”和“县域工业突破工程”，转变发展方式，推进产业结构调整和优化升级。围绕培育5家百亿元企业的目标，今年力争全市新增自治区百强企业4家，新培养产值超300亿元的企业1家，新增产值超10亿元企业5家，新增产值超亿元企业20家以上。

着力壮大临港工业。以钦州港经济开发区升格为国家级经济技术开发区为契机，加快完善提升开发区的功能和形象，强化港口和大企业带动作用，大力发展石化、造纸、能源和物流业，拉长产业链，形成临海产业集群。围绕加快建设临港石化基地，全力推进石化产业园公共管廊、供热供气、路网、双回电源以及石化码头、石化仓储设施等基础设施建设；全力支持服务中石油1000万吨炼油厂达产、含硫原油装置加快建设，加快中石油二期和600万吨lng项目、中海洋300万吨沥青、玉柴石化二期等项目前期工作，创造条件尽快开工建设；开工建设广西中石油国际公司580万立方储备油库，力促中亚石化、玉柴溶剂油、泰兴石化等竣工投产，力争在引进大乙烯、大芳烃项目方面取得重大突破。围绕加快建设临港林浆纸基地，全力推进造纸产业园的基础设施建设，重点支持金桂林浆纸一期浆达产、纸工程加快建设，加快推进林浆纸二期、翔盛粘胶纤维项目前期工作。围绕加快建设能源产业基地，全力争取燃煤电厂二期、石化产业园热电联供项目获批并开工建设。围绕加快建设临港粮油产业基地，推动中粮120万吨和汇海100万吨大豆加工项目竣工投产。加快推进修造船等项目建设。

着力培育发展战略性新兴产业。在加快推进河东电子工业园区建设的基础上，开展自治区级高新技术产业开发区的申报，编制好战略性新兴产业发展规划。加快河东电子工业园一期路网等设施建设，启动二期土地征拆工作。支持宇欣电子、福晟电子、天山电子做大规模，力促清华同方数字电视、唯万电子建成投产，积极引进产业链长、产业层次高、带动力强的装备制造、汽车等产业，大力发展新能源、新材料、生物等新兴产业。

着力发展县域工业。以启动县域省级经济技术开发区的建设和申报为载体，以招商引资为突破口，推动县域工业规模化、园区化发展，加快灵山工业园区、浦北工业集中区、钦南黎合江工业园、钦北皇马工业园、南间经济区等一批县域特色园区和一批标准厂房的建设。支持桂合丝业、兴莱鞋业、兴力达鞋业、建华管桩、祥云飞龙等县域骨干企业扩大生产，灵山阿帕奇、台湾宝成、钦北新合力、钦南钒钛铁分离等项目建成投产。

全力完成国企改制“三年攻坚计划”，大力支持企业技改，改造提升传统优势产业。

（三）坚定不移加快建设海湾新城，全力提升城市品质

以承办广西第十二届运动会为契机，深入开展“迎区运百项城建工程大会战”，畅通城市路网、完善市政设施、提升城市功能，充分展示钦州新形象。计划安排项目212项，总投资277.44亿元，完成年度投资49.99亿元。

完善城市规划设计。完成保税港区新城、市区旧城改造等城市专项规划和三娘湾旅游度假区控制性详规，完善滨海新城控制性详规及重点地段城市设计等新城各层次规划。完成新一轮县城总体规划修编，开展村镇体系规划、乡镇总体规划，推进县城和建制镇加快建设。

完善路网，拉开城市框架。重点抓好“一环六横六纵七通道”等一批路网项目建设，建成北部湾大道北段、环城西路（火车西站至钦州湾大道）、蓬莱大道北延长线、扬帆大道北延长线、富民路、兴桂路，继续推进环城北路、环城东路、子材东大街、南珠东大街，开工北部湾大道二期、钦江五桥（环城北路桥）、北部湾大道立交桥等，完成环城南路西段（金海湾西大街至钦江大桥）、钦江六桥（环城南路桥）等一批项目前期工作。

加快推进滨海新城等新区开发建设。突出抓好白石湖中央商务区、沙井岛休闲旅游区、辣椒槌生态区开发建设。推进白石湖中央商务区“一环三纵三横”主干道路及中央景观大道一期、白石湖水系景观工程等建设。建成北部湾大道一期、扬帆大道南延长线等骨干路网，全力推进已开工的北部湾国际大酒店等23个项目，新开工城市文化广场、规划展示中心、北美商贸城、人才孵化基地等一批标志性项目，滨海新城初现雏形。加快推进钦州港区行政商务中心建设。全面启动钦江、茅尾海综合整治工程。加快一批农民安置小区的建设。

加快完善中心城区功能。完成“一江两岸”精品示范工程；如期竣工市体育中心体育场、体育馆、游泳馆等区运会六大场馆；完善公交线网布局，加快一批公交站场和便民候车亭、停车场的建设，加大公交车、出租车运力投放和管理，方便市民出行；抓好钦州城区地震应急避难场所和城市备用水源工程建设。加快建设市第二水厂、河东污水处理厂和工业固体废弃物填埋场，开工垃圾焚烧发电项目。

着力提升城市管理水平。围绕创建国家生态园林城市目标，大力实施“园林生活十年计划”。进一步理顺城区管理体制，完善两级政府、三级管理的体制机制。积极推进“数字城管”建设，加强城市管理综合治理，深入实施“城乡清洁工程”和“城乡风貌改造”，规范完善城市标识标牌，开展以治理“五乱”为重点的市容环境整治活动，实施城市绿化、美化、亮化工程，改善城市景观，打造一批迎区运会景观精品线路和精品片区，努力创建自治区卫生城和文明城。

（四）坚定不移提升农业产业化水平，推动现代农业和新农村建设取得新成效

坚持“工业反哺农业，城市支持农村”和“多予、少取、放活”方针，加大城乡统筹力度，大力发展现代农业，推动城乡资源互动，促进农业发展和农民增收。

抓好“米袋子”和“菜篮子”。落实支农惠农政策，稳定粮食生产，严格耕地保护和基本农田保护，力争粮食总产稳定在106万吨以上。加快建设无公害蔬菜基地，扩大秋冬蔬菜种植规模。大力发展肉禽蛋奶等规模化生产基地。建立健全农产品质量安全体系，确保食品安全。推进农贸市场、超市与产地、农户的对接，降低流通成本，维持价格平稳。

加大对农村的基础性投入。加强新农村规划建设，加快推进病险水库除险加固、海河堤、大中型灌区节水改造、小型农田水利设施、农村安全饮水、农村公路和农村环境整治等，加快推进农业机械化，改善农业生产生活条件。完成26座列入规划的病险水库除险加固，解决25万农村人口饮水安全问题，实现85%的行政村通混凝土公路。

实施“特色农业提升工程”。大力发展荔枝、香蕉、火龙果、黄瓜皮、奶水牛、大蚝、对虾、名贵龟鳖、速丰林、花卉等特色农业，打造特色农业品牌。加快新技术、新成果、新品种的推广应用。支持桂合丝业、宇峰、百强、欧亚等农业产业化重点龙头企业发展，鼓励支持发展农村专业合作组织，建设一批农产品专业基地镇、基地村。实施“万元增收”工程，提高家庭经营性收入。全面实施“绿满八桂”造林绿化工程，完成灵山县、钦南区林改主体改革验收，推进配套改革。

（五）坚定不移提升服务业发展水平，推动第三产业发展上新台阶

以实施“东盟商贸物流基地工程”为抓手，加快发展现代服务业，与制造业共同推动钦州经济转型升级。

加强规划和政策引导。全面实施服务业发展规划，进一步完善文化、旅游等专项规划，启动专业批发市场、商业网点布局、专业街区等专项规划编制工作，出台支持和鼓励第三产业发展的政策措施，促进现代服务业快速健康发展。

加快推进一批商贸物流基础设施建设。重点加强保税港区、白石湖中央商务区、钦州港行政商务中心等各具特色的服务业聚集区、功能区建设。推进沃尔玛购物中心等一批商业网点建设，加快中国－东盟商贸城、中国－东盟（钦州）大型农产品大市场等专业市场建设，改造提升老城区商业环境。

鼓励发展连锁经营、物流配送、电子商务等现代流通方式，发展超市、专卖店、便利店、电视购物等新兴业态，促进传统商贸流通业升级。鼓励发展微型商业企业，培养壮大经济新力量。进一步实施“万村千乡”市场工程，不断完善城乡流通网络。认真贯彻落实国家家电以旧换新政策，继续做好家电下乡工作。切实做好稳定物价保证供应工作。

提升旅游业发展水平。依托滨海旅游资源优势和茅尾海国家海洋公园、中华白海豚之乡、千年古陶都的品牌，积极引进有实力的大企业集团开发建设大型高档旅游度假区、主题公园、特色文化街区，打造茅尾海、七十二泾、三娘湾、八寨沟、刘冯故居、五皇山、六峰山、大芦村、世外茶园等一批旅游精品，着力打造具有钦州特色旅游节庆文化品牌，加大特色旅游产品研发力度。加快宾馆酒店升星改造。加强旅游服务和宣传推介，进一步拓展国内外旅游市场。

大力发展现代物流业。加快钦州港综合物流加工区、浦北物流中心、钦州港大型煤炭配送基地等一批物流项目建设，加快引进实力强、服务优的现代物流企业，发展壮大物流业。依托石化、粮油等优势产业，整合物流资源，推进物流基础设施建设，发展和完善包括仓储、包装、运输、配送、分拣、加工等在内的物流服务体系，打造区域性物流平台。

（六）坚定不移深化改革，扩大开放，不断增强加快发展新动力

坚持改革创新。继续争取“整体用海报批”；加快推进城乡建设用地增减挂钩和集体建设用地流转，完善征地拆迁机制，全面推行征地拆迁公寓式安置；鼓励和支持中小企业信用担保机构建设；做大做强市开投集团、临海公司、滨海置业集团，创新融资模式，开拓金融新产品，积极培育上市公司；充分发挥港口物流等四个战略领导小组的作用，积极引进拔尖人才和高技术人才，为经济发展提供智力支撑。

理顺园区管理体制和开发机制，吸引社会、民间资本参与园区建设，形成园区多元投入机制，力争做到区内事情区内办。理顺城区管理体制，调动城区管理城市的积极性和主动性。继续实行“阳光和效能重点建设岗位”制度，全面推进平衡计分卡绩效考评，加强软环境建设，提高行政效能。

深化开放合作。推进出口市场多元化，巩固东盟、欧美、日韩等主体市场，拓展非洲、中东、大洋洲、南美洲等新兴市场，扩大电子、轻工、机械、纺织品、农产品出口，提高产品出口附加值；充分利用保税港区、进口资源加工区、电子工业园区等平台，引进加工贸易企业，发展壮大加工贸易业，努力扩大外贸进出口。以钦州（湖南）临港工业园、台湾石化园区、韩国产业园、马来西亚产业园等产业合作区为平台，积极参与国际区域合作，努力推进跨国界跨区域合作。

完善专业招商服务团队工作机制，建立健全招商会商、项目跟踪、项目代办等机制，创新招商方式，推行委托代理招商，扩大以商招商，推进网上招商，鼓励全民招商。开展产业链招商，力争在乙烯、芳烃等石化项目、保税物流项目、汽车零配件和装备制造项目、电子项目上有重大突破。完善招商引资考评机制，健全考核奖惩办法。在拓宽外资领域、优化外资机构、产业投资引导、管理体制改革、优化投资环境等方面下功夫，努力开创我市利用外资新局面。

（七）坚定不移强科教，适应钦州跨越发展的新需要

大力实施科教和人才强市工程，促进文化大繁荣大发展，提升钦州发展软实力。

推动科技创新。大力培育高新技术企业，促进高新技术产业发展。加强科技平台建设，促进科技与经济相结合；加快高新技术服务中心建设，加大重点实验室的扶持力度；围绕支柱产业和特色产业，实施科技攻关工程，促进科技成果转化。加强知识产权保护，实施科技富民强县试点；加强科普工作。

优先发展教育。积极申办北部湾大学，大力发展职业教育。实施学前教育三年行动计划，新建5所公立幼儿园和一批民办幼儿园。实施教育布局结构调整规划，加快市实验小学、市外国语学校、市四中、钦州港中学等4所中小学新校区建设，继续实施中小学校舍安全工程，对一批农村义务教育薄弱学校校舍进行改造，巩固义务教育成果，逐步实现“好上学、上好学”。健全家庭经济困难学生资助体系，开展教师周转房建设试点。

促进文化大繁荣。加快发展文化事业和文化产业，扶持发展坭兴陶产业和文化创意等新兴产业，加强农村公共文化服务项目建设。建设广西北部湾博物馆、市文化科技活动中心、千年古陶城、数字电影院等项目，以国家级爱国主义教育基地的标准加快刘永福旧居维修和提升工作。

全力承办好区运会。以承办广西第十二届运动会为契机，积极开展群众性精神文明创建活动，大力推进文明城、卫生城和生态园林城市创建活动，促进全民健身运动广泛开展，提升全市文明水平。高水平、高标准做好运动会各项筹备工作，确保以一流的场馆设施、一流的组织服务、一流的竞赛成绩、一流的城市形象当好东道主，努力把区运会办成平安、祥和、文明、精彩的体育文化盛会，展现钦州风采。

（八）坚定不移惠民生，满足群众构建和谐社会的新期待

实施“三业”行动富民工程。建立健全劳动者自主择业、市场调节就业和政府促进就业相结合的机制，深入开展就业、创业、兴业“三业”行动，开发更多的公益性岗位，鼓励农村劳动力就地就近转移就业；完善就业创业服务体系，加强职业培训特别是农民工的就业培训，突出抓好下岗失业人员、失地农民、失海渔民、困难大学毕业生等弱势群体的就业援助工作，支持自主创业和自谋职业，促进社会充分就业。加强劳动执法，保障劳动者权益。

加快发展医疗卫生事业。完成医药卫生体制改革五项重点任务，推进基层医疗卫生机构综合改革，100%的基层医疗机构实施基本药物制度。继续推动基本公共卫生服务均等化和实施防治艾滋病攻坚工程。

建立健全社会保障和救助体系。建立健全社会保险、社会救助、社会福利等社会保障体系。扩大社会保障覆盖面，扩大新农保试点；落实好被征地农民、失海渔民社会保障政策，加快解决国有困难企业退休人员养老保险和医疗保险；加快老年人服务基础设施建设，大力发展居家养老服务。加快儿童福利机构建设，建立孤儿保障制度；扩大保障性住房建设，实施农村危房改造工程。

协调发展社会各项事业。切实抓好人口和计划生育工作。积极推动生态文明建设，加快节能减排工程；完善节能减排工作机制，深入实施全民节能减排行动，积极发展循环经济。落实各项优抚安置政策，支持部队和国防建设，巩固和发展军政军民团结。继续做好工会、共青团、妇联、外事、侨务、民族、宗教、气象、防震减灾、水文、档案、地方志、老龄等工作。

继续实施为民办实事工程。从解决群众最关心、最直接、最紧迫的问题入手，投入26.01亿元，办好10项惠民工程，让人民群众共享发展成果（具体项目见附表）。

保持社会和谐稳定。推行安全生产“一岗双责”，全面加强企业安全管理，落实企业安全生产主体责任，健全应急救援体系，促进安全发展。完善社会治安防控体系，依法打击违法犯罪活动。强化社会治安综合治理，深入开展平安和谐钦州建设活动，启动和实施“六五”普法，不断增强公民法律意识，及时妥善化解社会矛盾。强化食品药品监管，保障群众的健康和安全。

（九）坚定不移培植壮大财源，力争财政保障能力有新提高

加强地方财源建设。去年财政总收入快速增长，但一般预算收入增幅不大，可用财力不多，收支平衡压力仍然很大。因此，必须继续加快重大基础设施项目建设，抓好石化、冶金、矿产加工、能源、制糖、粮油加工等优势行业、重点企业促产达产扩产的同时，要积极发展现代服务业，培育新的财源增长点，千方百计增加地方可用财力，努力扭转财政总收入增长快，地方可用财力小的局面。做好重大税源的跟踪管理，实行目标量化管理，做到应收尽收。

统筹财政支出。坚持厉行节约,优化支出结构，压缩一般性支出，出国经费、公务车辆购置及公务接待经费等原则上实行零增长。保障为民办实事、扩大内需消费、重点税源、困难群众基本生活等方面支出，继续推进事业单位绩效工资改革，提高财政供养人员收入水平，逐步缩小市、县、区收入差距。继续加大对“三农”、教育、卫生、文化和节能减排工程等项目的投入。加强政府债务管理和信用建设。

（十）坚定不移加强政府自身建设，努力提高政府的执行力、公信力和创新力

推动钦州科学发展，人民群众寄予厚望，政府责任重大。我们要全面推进管理创新，努力建设让人民满意的政府、让人民信赖的政府、让人民放心的政府，提高政府的执行力和公信力。要不断改进政府管理与服务方式，加强电子政务建设，推进联合审批，简化规范审批事项，提高行政效能。开展好“好干部、善执行”活动，提升政府行政执行力和创新力。进一步提高依法行政能力和水平，建立健全科学民主决策机制，增强决策透明度和公众参与度。自觉接受人大的法律和工作监督，主动接受政协的民主监督，认真听取各民主党派、工商联、无党派人士和人民团体的意见，高效办理人大议案、政协提案。畅通倾听民意、集中民智、维护民利的渠道，主动接受新闻舆论和群众监督。加强党风廉政建设，完善惩治和预防腐败体系，加强权力运行的监督，加大专项治理和纠风工作力度，着力从源头上预防腐败。坚持艰苦奋斗，厉行节约，坚决制止奢侈浪费。加强监察审计、政务督查和限时督办，严肃查处有令不行、有禁不止和失职渎职等行为，加大行政问责力度，塑造风清气正的政务环境。要讲政治、当公仆、干实事，努力创造新的业绩，决不辜负全市人民的期望！

各位代表，让我们倍加珍惜上下齐心的和谐局面，倍加珍惜来之不易的发展势头，倍加珍惜难能可贵的发展机遇，在市委的正确领导下，满怀激情，凝心聚力，锐意进取，扎实工作，为开创我市科学发展、和谐发展、跨越发展新局面，夺取全面建设小康社会新胜利，谱写钦州人民美好生活新篇章而努力奋斗！
